# C-Shaped Frontalis Muscle Flap Suspension for Congenital Blepharoptosis: A Retrospective Analysis of Outcomes in Moderate to Severe Cases

**DOI:** 10.1055/a-2640-3975

**Published:** 2025-09-01

**Authors:** Minh Ngoc Pham, Rong-Min Baek, Anh Mong Vu Luong

**Affiliations:** 1 Craniofacial and Plastic Surgery Department, 108 Military Central Hospital, Hanoi, Vietnam; 2 Department of Plastic and Reconstructive Surgery, Seoul National University Bundang Hospital, Republic of Korea; 3 College of Health Sciences, VinUniversity, Hanoi, Vietnam

**Keywords:** moderate and severe blepharoptosis, frontalis muscle flap suspension, poor levator function

## Abstract

**Background:**

While various surgical techniques have been developed for blepharoptosis correction, the frontalis suspension technique is commonly applied in cases of poor levator function or prior surgical history. This study evaluates the efficacy of a modified frontalis muscle flap suspension technique in achieving satisfactory outcomes for severe or recurrent blepharoptosis with poor levator function.

**Methods:**

A retrospective study conducted from January 2014 to January 2017 reviewed the medical records of 47 patients with a mean age of 17.3 ± 9.17 years at 108 Military Central Hospital, Ha Noi, Vietnam. These patients were diagnosed with moderate to severe blepharoptosis (marginal reflex distance 1, MRD1 0–2 mm) with poor levator function (<4 mm) and underwent modified C-shaped frontalis muscle flap suspension. The outcomes were measured by the sum of functional and cosmetic grading scales at 6- and 12-month follow-ups. Postoperative complications were also noted.

**Results:**

There are 40 patients (85.1%) who have unilateral ptosis and 7 (14.9%) who have bilateral ptosis. Forty-seven patients (87%) had severe ptosis, while seven (13%) had moderate ptosis. A history of frontalis sling surgery was present in 38.9% of patients. At the 12-month follow-up, 37 patients (78.7%) had good outcomes, 9 patients (19.1%) had fair outcomes, and 1 patient (2.1%) had poor outcomes that underwent surgical revision.

**Conclusion:**

Our analysis of the modified C-shaped frontalis muscle flap suspension technique demonstrates its efficacy in treating moderate and severe blepharoptosis, particularly in cases with poor levator function and prior surgical history.

## Introduction

Blepharoptosis is characterized by an abnormal drooping of the upper eyelid margin in primary gaze, which covers the upper pole border of the cornea by approximately 1 to 2 mm. 1 It is classified as either congenital or acquired, and is typically unilateral (70%) but can also be bilateral. It is associated with disease in one or more extraorbital muscles or with other systemic diseases. 2 Blepharoptosis affects not only the visual aspect but also the visual function, as it obstructs the visual axis. Lee et al. (2018) studied 2,328 patients who underwent blepharoptosis surgery at a Korean hospital from 1991 to 2014 and found that 1,815 (78%) had congenital blepharoptosis, while 513 (22%) had acquired blepharoptosis. Simple congenital blepharoptosis was the most prominent type (73.7%), while aponeurosis was the most common cause of acquired blepharoptosis. 3

Surgical intervention is the primary treatment for blepharoptosis. These procedures involve either improving the function of the levator muscle by shortening the levator fascia or employing the frontalis muscle as the driving force to passively suspend the upper eyelid. Levator aponeurosis shortening is indicated for treating mild to moderate blepharoptosis because it maintains the upper eyelid's natural contours with minimal tissue distortion, providing visual benefits. This approach is ineffective for ptosis with poor levator function. 4 5

Frontalis sling techniques with alloplastic suspension material (non-absorbable, silicone thread) or autologous (fascia lata) are indicated for severe blepharoptosis with poor levator function. This procedure is straightforward, with a short operation duration; however, the recurrence rate was relatively high compared to other methods, and complications such as lagophthalmos and keratitis may occur. 6 7

The conjoint fascial sheath (CFS), first described by Dr. Whitnall in 1932, is a fibrous tissue between the levator muscle and the superior rectus. It was first used to correct blepharoptosis in 2002 by Holmström and Santanelli, and in 2019, Zhou et al. introduced a minimally invasive CFS suspension technique for treating mild and moderate ptosis. 8 9 10 The CFS has emerged as a new suspension tissue for treating ptosis, but this technique has its drawbacks. Resection of the levator can disrupt the muscle and affect eye closure recovery, may lead to postsurgical infraduction, and there is limited evidence supporting its effectiveness in cases of severe ptosis. 11

The frontalis muscle flap suspension technique essentially substitutes the activity of the levator muscle with the contraction of the frontal muscle, which can be indicated in severe blepharoptosis with poor levator function. Since it employs direct dynamic movement of the frontal muscles, this method is the most efficient and physiological, and it can circumvent the limitations of frontalis sling techniques. Since Fergus performed the first frontalis muscle flap suspension in 1901, this technique has been gradually modified and extensively applied. 12 In 1982, Song and Song successfully applied the modified L-shaped frontalis muscle flap suspension on 30 Asian patients, achieving good and lasting cosmetic and functional results. 13 In 1993, Han and Kang modified the technique to create a tripartite frontalis muscle flap transposition, which provided an even distribution of upward pull on the tarsus without tenting the lid margin. 14 In 2010, Lai et al. used the frontalis-orbicularis oculi muscle flap shortening technique to correct blepharoptosis with good biomechanics in place of the traditional frontalis muscle sling. 15 However, the L-shaped flap has several disadvantages, including increased tensile force at the most medial suture of the flap onto the tarsal plate. Additionally, if the distance between the eyebrow and the tarsal plate is long, it further increases tensile force and requires additional muscle resection to pull the flap for suturing. The tripartite frontalis flap can cause muscle damage due to muscle separation into three segments, potentially leading to fibrosis over time and weakening its elevating force. Therefore, we propose a modified C-shaped frontalis muscle flap that utilizes rotational movement to enhance flap mobility, reduce tensile force at the most medial suturing point, minimize muscle resection, and preserve frontalis muscle function.

We conducted this study to assess the clinical effectiveness of a C-shaped frontalis muscle flap suspension for treating moderate and severe blepharoptosis in patients with poor levator function.

## Methods

### Patients

A retrospective study reviewed the medical records of patients who underwent ptosis correction surgery performed by the same surgeon in the Department of Maxillofacial Surgery and Plastic Surgery at 108 Military Central Hospital between January 2014 and January 2017. The inclusion criteria were as follows: (1) congenital moderate to severe blepharoptosis (MRD1 0–2 mm) with poor levator function (<4 mm), (2) intact frontalis muscle, (3) positive Bell's sign, and (4) absence of systemic or progressive diseases. Patients with concurrent oculomotor paralysis, frontalis muscle injury, a negative Bell's phenomenon, or systemic diseases that contraindicated anesthesia were excluded from the study. In these excluded patients, surgery was not performed due to the risk of postoperative corneal abrasion caused by limited ocular movement.

The studied data include age, gender, unilateral or bilateral ptosis, etiology, past history of frontalis sling surgery, types of anesthesia, postoperative functional and cosmetic evaluation ( Table 1 ) at 6-and 12-month follow-ups, and surgical complications. We obtained informed consent and permission for the publication of clinical photographs from all patients.

**Table 1:** Postoperative grading system for evaluating frontalis muscle flap function

Functional criteria a	0	1	2	3	4
Severity of ptosis (MRD1, mm)	Severe (0–1)	–	Moderate (1–2)	Mild (2–3)	Normal (3–5)
Levator function (mm)	Poor (≤1)	–	Fair (1–2)	–	Good (>2)
Lagophthalmos (mm)	Severe (>4)	–	Mild (0.5–4)	–	None (≤0.5)
Palpebral fissure height (mm)	Poor (≤1.5)	Fair (1.5–2.5)	Good (>2.5)	–	–
Forehead skin sensation	Poor	Fair	Good	–	–
Cosmetic criteria b					
Lid contour in primary gaze	Unnatural	Natural	–	–	–
Lid contour in upward gaze position	Unnatural	Natural	–	–	–
Eyelid crease	Poor	Good	Excellent	–	–
Eyelid scar	Obvious	Subtle	–	–	–
Eyebrow scar	Obvious	Subtle	–	–	–
Patients's satisfaction	Unsatisfied	Acceptable	Satisfied	–	–

Abbreviation: MRD1, margin reflex distance 1.

a Functional criteria with total points of 16, categorized as good (>11), fair (8–11), and poor (<8).

b Cosmetic criteria with total points of 8, categorized as good (>4), fair (3–4), and poor (<3).

### Surgical Technique

**Fig. 1:**
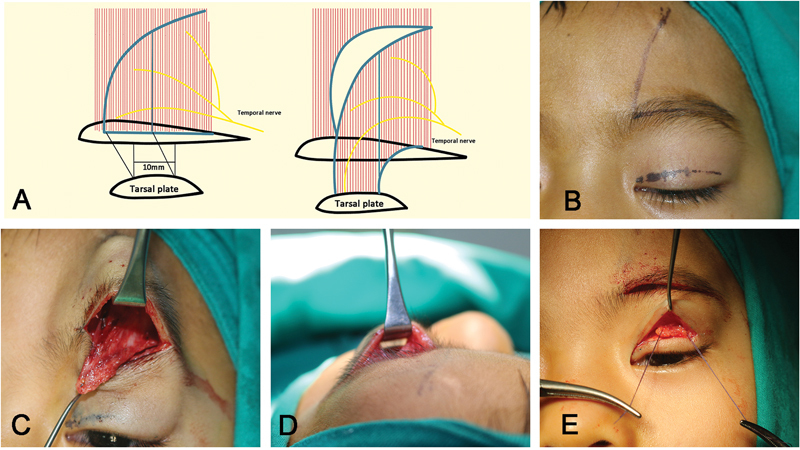
C-shaped frontalis muscle flap procedures **(A)** . Eyebrow incision, forehead muscle flap, and a new eyelid crease line were designed **(B)** . Subcutaneous dissection was performed on the anterior surface of the frontalis muscle, and a C-shaped frontalis muscle flap was created by cutting over the interdigitated part of the frontalis muscle and orbicularis oculi muscle (IFO) **(C)** . A tunnel was formed that extended from the upper eyelid to the eyebrow **(D)** . The frontalis muscle flap was sutured to the middle of the pretarsal plate at three different spots using a non-absorbable material **(E)** .

Our surgical technique is a modification of the method originally proposed by Song and Song (1982). 13 General anesthesia is used on young children, while local anesthesia is favored for adult patients. In cases of unilateral ptosis, surgical markings were made based on the height of the contralateral upper eyelid, and in cases of bilateral ptosis, markings were made according to the physiological height of 7 to 8 mm. The markings include an eyebrow incision, the shape and position of the harvested frontalis flap, and a new eyelid crease line ( Fig. 1B ). An incision along the designed markings in the eyebrow was made, and subcutaneous dissection was performed to expose the prefrontalis muscle. Compared to Song and Song's technique, in which the flap is designed as an L-shape with a horizontal incision along the lower margin of the frontalis muscle and a vertical incision forming a right angle, 13 our approach modifies this by shaping the incision the frontalis flap into a C-shape with a tip flap angle of 60 to 70 degrees ( Fig. 1A ). This modification facilitates rotational movement, enhancing the flap's mobility to the tarsal plate while reducing tensile forces on the sutures, particularly at the most medial suturing point.

The inferior border of the frontalis muscle at the interdigitated part with the orbicularis oculi muscle was resected along the entire length of the eyebrow line. Then, dissection was performed to expose the posterior aspect of the frontalis muscle. The medial border of the frontal muscle was cut in a C-shaped flap inferiorly to superiorly, medially to laterally, and parallel to the frontal muscle fibers in an outward curve. To prevent damaging the supraorbital neurovascular bundle during this step, the supraorbital nerves must be meticulously identified, and the incision for the frontalis muscle flap should be made approximately 5 mm medial to the supraorbital foramen. The frontalis muscle flap was created with a medial head that freely descends toward the eyelids ( Fig. 1C ). The extent of the flap dissection was based on the need for the sling. After completing the flap, an incision was made in the upper eyelid skin as designed, followed by subcutaneous dissection to expose the pretarsal plate. Orbital fat was removed to facilitate eyelid mobility. A tunnel was formed beneath the septum by subcutaneous dissection that extended from the upper eyelid to the eyebrow ( Fig. 1D ). The frontalis muscle flap was then drawn through the tunnel, and sutured to the middle of the pretarsal plate at three different spots using a non-absorbable material ( Fig. 1E ). The position of the frontalis flap attachment is at the middle of the tarsal plate to prevent the risk of entropion or ectropion, which can occur if the attachment is positioned higher or lower than the central point.

After suturing, the height of the palpebral fissure was measured to determine whether it had achieved the normal physiological height of 8 to 9 mm. The height can be adjusted by trimming the tip of the frontalis muscle flap. If there is any excess skin, 1 to 3 mm of it might be excised on the pathological eye. After hemostasis was achieved, the eyebrow incision was closed in two layers, and a drainage strip was placed. The upper eyelid crease was closed. A modest bandage compression was applied to the forehead. Antibiotics, pain relievers, and antiedematous medications, as well as eye ointments, were provided postoperatively. Cold application was used for the first 24 hours after surgery to minimize the inflammation and swelling. The sutures were removed after a week following surgery.

### Postoperative Evaluation

An evaluation was performed at 6 and 12 months postoperatively. There is no standardized grading scale for postoperative evaluation in ptosis correction studies, and while some studies include grading scales, they incorporate fewer variables and primarily assess them independently. Therefore, adapting from previous studies, 16 17 18 we developed a grading system with multiple variables to assess postoperative outcomes of blepharoptosis correction, integrating both functional and cosmetic evaluations ( Table 1 ). The functional evaluation included ptosis severity, levator function, lagophthalmos, palpebral fissure height, and forehead skin sensation, and the results were graded as good (>11), fair (8–11), or poor (<8) for a total of 16 points. MRD1 is the distance, measured in millimeters, between the upper eyelid margin and the corneal light reflex. Levator function is assessed by measuring the distance the eyelid travels from downgaze to upgaze while keeping the frontalis muscle inactive at the brow. Lagophthalmos is the height of the remaining palpebral fissure when the eyelids are closed. Palpebral fissure height is the vertical distance between the open eyelids. Forehead skin sensation is assessed by comparing it to the opposite area using both pinprick and cold sensation tests. The cosmetic evaluation included lid contour in primary gaze, lid contour in upward gaze position, eyelid crease, eyelid scar, eyebrow scar, patient's satisfaction, and the results were graded as good (>4), fair (3–4), or poor (<3) for a total of 8 points. Lid contour is evaluated by observing for a natural, symmetrical curve without any peaking or flattening in both the primary gaze and upward gaze positions. The eyelid crease is graded as follows: excellent (2) for symmetry without obliteration, good (1) for a mild asymmetry with slight obliteration, and poor (0) for a complete loss of the lid crease. Eyelid and eyebrow scars are considered obvious if they are visibly noticeable. Functional and cosmetic evaluations are performed by both the patient and independent plastic surgeons who were not involved in the treatment. Since cosmetic criteria can be subjective, two plastic surgeons independently evaluated randomly arranged patient photographs using the same criteria to assess both functional and cosmetic outcomes. The final score was calculated as the average of the two evaluations to ensure reliability. The overall outcome would be calculated by the sum of functional and cosmetic evaluation and be graded as good (>15), fair (11–15), and poor (<11) outcome. Any postoperative complications were also noted.

## Results

From January 2014 to January 2019, 47 patients with a total of 54 lids who had moderate to severe blepharoptosis with poor levator function had their ptosis corrected. The ages of the patients range from 4 to 33 years, with a mean of 17.3 years. Regarding past surgical history, 21 eyes (38.9%) had a history of frontalis sling surgery, while 33 eyes (61.1%) were undergoing surgery for the first time. There were 40 patients (85.1%) who had unilateral ptosis and 7 (14.9%) who had bilateral ptosis. The location of ptosis comprises 20 right lids (37%) and 34 left lids (63.0%). Most patients are female (61.7%), with 38.3% being male. Preoperative assessment of ptosis severity showed severe ptosis in 47 patients (87%) and moderate ptosis in 7 patients (13%). Preoperative examination of levator function revealed poor function in 52 patients (96.3%) and moderate function in 2 patients (3.7%). The preoperative features of patients are shown in Table 2 .

**Table 2:** Preoperative patient features

Variable	*N*	Percentage
Gender		
Male	18	38.3
Female	29	61.7
Past blepharoplasty history, *n* (%)		
None	33	61.1
≥1 times	21	38.9
Distribution of ptosis, *n* (%)		
Unilateral	40	85.1
Bilateral	7	14.9
Location of ptosis, *n* (%)		
Right	20	37
Left	34	63
Degree of ptosis, *n* (%)		
Severe	47	87
Moderate	7	13
Levator function, *n* (%)		
Poor	52	96.3
Moderate	2	3.7
Age (years), mean ± SD	17.34 ± 9.17 (4–33)	

Abbreviation: SD, standard deviation.

**Fig. 2:**
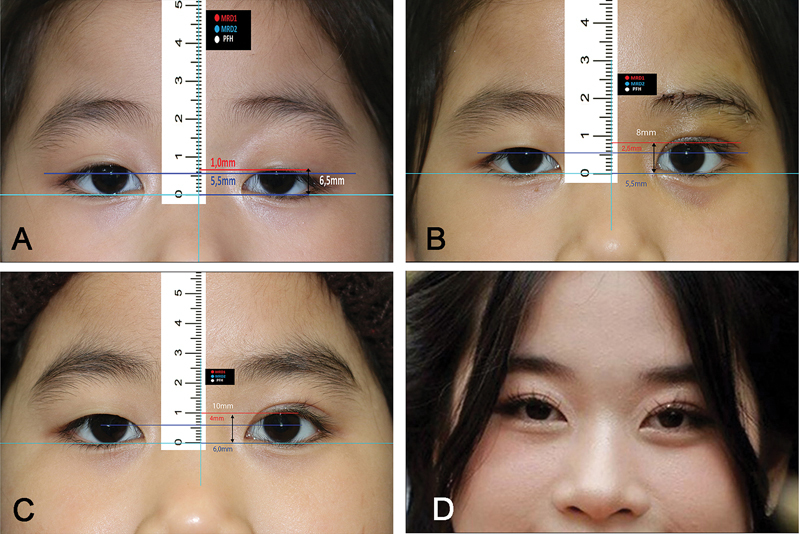
Pre- and postoperative pictures of MRD1 and MRD2 changes in a 9-year-old child who had congenital blepharoptosis in her left eye. Preoperative anterior view, MRD1 1 mm **(A)** . Six-month postoperative anterior view, MRD1 2.5 mm **(B)** . Twelve-month postoperative anterior view, MRD1 4 mm **(C)** . Five-year postoperative anterior view **(D)** . MRD, marginal reflex distance.

**Fig. 3:**
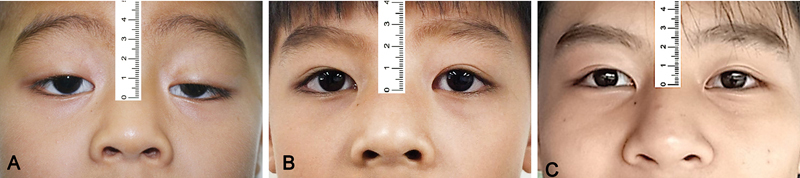
An 8-year-old boy with severe congenital blepharoptosis in the left eye and moderate blepharoptosis in the right eye was treated with bilateral frontalis muscle flap suspension. Preoperative photograph **(A)** . One-year postoperative photograph in primary gaze **(B)** . Five-year postoperative photograph in primary gaze **(C)** .

Preoperative MRD1 ranged from −1.5 to 1 mm with an average of 0.009 ± 0.6 mm, postoperative MRD1 after 6 months ranged from 2 to 5.5 mm with an average of 3.63 ± 0.77 mm, and postoperative MRD1 after 12 months ranged from 2.0 to 5.5 mm with an average of 3.63 ± 0.77 mm. Preoperative palpebral fissure height ranged from 3.0 to 7.0 mm with an average of 5.59 ± 0.68 mm, postoperative palpebral fissure height after 6 months ranged from 7.5 to 11.0 mm with an average of 9.24 ± 0.85 mm, postoperative palpebral fissure height after 12 months ranged from 7.5 to 11.0 mm with an average of 9.02 ± 0.89 mm. Regarding the overall outcome after 12 months, 37 patients (78.7%) had good outcomes, 9 patients (19.1%) had fair outcomes, and 1 patient (2.1%) had poor outcomes. Lagophthalmos was observed in more than half of the patients within 1 week, but most of them recovered. Ten patients (18.5%) still had lagophthalmos after 6 months, and four patients (9.3%) had it after a year. In terms of lid lag postoperatively, there are 16 patients (29.6%) at 6 months and 5 patients (9.3%) after 12 months. No forehead sensation loss was noticed in all of the cases after 12 months, while a decrease in forehead sensation occurred in 8 of 54 eyes (14.8%). There are no cases of patients experiencing severe complications such as hematoma, conjunctival prolapse, or eyebrow asymmetry. In our study, three patients underwent a follow-up period of 4 to 5 years and demonstrated favorable outcomes. The surgical outcomes and complications are shown in Table 3 and Figs. 2 and 3 .

**Table 3:** Postoperative evaluation

Variable	Preoperative	Postoperative	
		6 months	12 months
MRD1 (mm), mean ± SD (range)	0.009 ± 0.60 (−1.5–1.0)	3.63 ± 0.77 (2.0–5.5)	3.45 ± 0.80 (2.0–5.5)
Palpebral fissure height (mm), mean ± SD (range)	5.59 ± 0.68 (3.0–7.0)	9.24 ± 0.85 (7.5–11.0)	9.02 ± 0.89 (7.5–11.0)
Lagophthalmos, *n* (%)	–	10 (18.5%)	4 (7.4%)
Lid lag, *n* (%)	–	16 (29.6%)	5 (9.3%)
Overall outcome at 12 months, *n* (%)	Good		37 (78.7%)
	Fair		9 (19.1%)
	Poor		1 (2.1%)
	Mean point ± SD		15.13 ± 2.03

Abbreviation: SD, standard deviation.

## Discussion

Frontalis muscle flap suspension is a technique that directly substitutes the forehead muscle for a weak or non-functional levator muscle. It is recommended for cases with moderate to severe blepharoptosis with poor levator function (<4 mm). 19 20 21 22 The technique has a variety of advantages, including direct application of traction of the frontalis muscle on the eyelids (tarsal plate), no need for fascia lata or alloplastic material which is easy to slip or break, and improved traction direction with the upper eyelid pulled toward the eyebrow instead of the eyeball.

CFS suspension has recently emerged as a new suspension technique for treating ptosis, offering several advantages over frontalis muscle suspension in efficacy, complication rates, and physiological movement. It achieves higher correction efficiency (90.70% vs. 71.88%) and fewer complications. 23 Additionally, CFS provides more natural eyelid movement. 23 While CFS suspension has shown promising results in correcting severe blepharoptosis, evidence supporting its effectiveness remains limited, warranting further studies. CFS suspension also has certain limitations, including the potential for infraduction due to partial involvement of the superior rectus muscle, which may increase the risk of corneal exposure and subsequent keratitis. 12 Moreover, in cases of poor levator muscle function, eyelid elevation may be suboptimal, further compromising postoperative outcomes. Currently, CFS is more suitable for mild to moderate ptosis, while frontalis suspension remains the most effective approach for severe blepharoptosis with poor levator function, particularly in patients with unilateral congenital ptosis. 24

Over the years, advancements in frontalis muscle flap techniques have significantly improved ptosis correction, focusing on enhancing eyelid elevation, reducing complications, and achieving better aesthetic outcomes. The initial use of a frontalis muscle flap by Fergus in 1901 marked the beginning of surgical innovations aimed at refining upper eyelid suspension. By 1982, Song and Song introduced the L-shaped frontalis muscle flap, which became widely adopted due to its ability to provide stable eyelid elevation. 13 Subsequent studies further validated its effectiveness, demonstrating high success rates in severe ptosis cases. 25 26 27 However, challenges of the L-shaped frontalis flap, such as excessive muscle resection and increased tension at the most medial suturing point, especially in cases of patients having a long distance between eyebrows and tarsal plate.

Building on these advancements, our approach using the C-shaped frontalis muscle flap offers several advantages. Unlike the L-shaped flap, which primarily relies on translational movement, the C-shaped flap features a tip flap angle of 60 to 70 degrees, enabling rotational movement that enhances mobility and adaptability to the tarsal plate, while also reducing tensile force at sutures, particularly at the most medial suturing point. This design not only improves functional outcomes but also reduces the length of frontalis muscle resection, thereby minimizing muscle injury and decreasing the risk of forehead sensory loss—a finding supported by our study, in which no patients reported sensory loss after 12 months with 8 out of 54 eyes (14.8%) experiencing a decrease in sensation.

Our study on 54 eyes (47 patients) found that the average age of patients with ptosis was 17.34 ± 9.17 years, with a range of 4 to 33 years, demonstrating that our approach is applicable across a wide range of ages, including children. This is similar to the average age in the studies by Li et al. (2016) 28 and Wang et al. (2017). 17 In our study, there is a chance that 38.9% of eyes have a history of frontalis sling surgery at least once and require surgery due to recurrence. In this group, the overall outcomes of cosmetic and functional were 16.9  ±  2.4, which were not statistically different from those of patients with no history of previous surgery (17.7 ± 2.6; *p* > 0.05). In these patients, the artificial-material sling, which had caused inflammation in the surrounding tissues, was removed and replaced with a frontalis muscle flap. This finding suggests our technique could be particularly beneficial for patients with a history of frontalis sling surgery. Two-thirds of our patients had left-eye blepharoptosis, and the other third with right-eye blepharoptosis. MRD1 improved statistically from 0.009 ± 0.60 mm preoperatively to 3.63 ± 0.77 mm at 6 months and 3.45 ± 0.80 mm at 12 months postoperatively. Our findings are comparable to those of Lai et al. (2013), who used an orbicularis oculi-frontalis muscle flap on 81 ptosis eyes (66 patients) with poor levator function. In their study, MRD1 increased from −1.6 ± 2.0 mm preoperatively to 3.3 ± 1.2 mm postoperatively. 18 In the study, the palpebral fissure height improved statistically from 5.59 ± 0.68 mm preoperatively to 9.24 ± 0.85 mm at 6 months and 9.02 ± 0.89 mm at 12 months postoperatively.

In terms of the overall outcomes at 12 months, the majority of patients had good results (78.7%) and fair results (19.1%), and only 2.1% of patients had poor results. Our findings are consistent with those found in other studies, such as that of Bagheri et al. (2012), 29 Hou et al. (2013), 25 and Li et al. (2016) 28 of 100% good and fair results. Bhiromekraibhak's (2010) study on 12 severe congenital ptoses (8 patients) using the orbicularis oculi-frontalis muscle flap showed good outcomes after 2 weeks. No complications were observed. 30 Our initial study followed patients for 12 months, but encouragingly, three patients followed for 4 to 5 years showed favorable outcomes. This suggests our technique may have lasting benefits, but a dedicated long-term study is warranted for confirmation. One patient in our study experienced a poor outcome at the 12-month follow-up. This is because the patient had congenital blepharoptosis concurrent with an orbital floor and lateral wall fracture, making the surgery more challenging. As a result, it was difficult to properly fix the flap, leading to an asymmetrical A-shaped eyelid crease and poor cosmetic outcome. In response, we implemented a surgical revision, including shortening the flap and adjusting the flap sutures to fix on the tarsal plate, resulting in a favorable outcome.

**Fig. 4:**
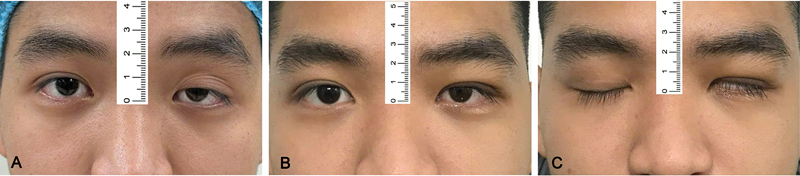
A 23-year-old male with severe blepharoptosis in the left eye was treated with frontalis muscle flap suspension. Preoperative photograph **(A)** . One-year postoperative photograph in primary gaze **(B)** . One-year postoperative photograph with eyes closed **(C)** .

**Fig. 5:**
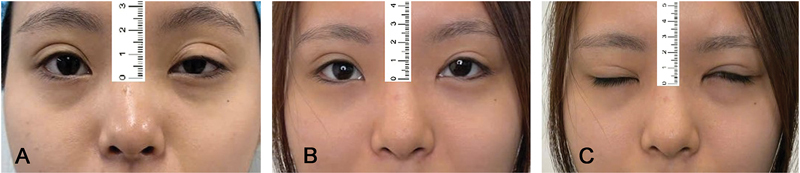
A 22-year-old female with severe blepharoptosis in the left eye was treated with frontalis muscle flap suspension. Preoperative photograph **(A)** . One-year postoperative photograph in primary gaze **(B)** . One-year postoperative photograph with eyes closed **(C)** .

Mokhtarzadeh and Bradley (2016) evaluated a cohort of 47 children with congenital ptosis and found that complications following surgery included lagophthalmos in 19/47 children (40.4%) and dry eyes in 3/47 children (6.4%). 31 In a study by Li et al. (2016) on 80 patients using a frontalis aponeurosis flap with 1 to 3 years of follow-up, it was found that 79 patients could totally close their eyes and obtain good functional results within 3 months. One patient had incomplete eyelid closure 6 months postsurgery and developed mild keratitis. 28 According to our findings, the percentage of patients having lagophthalmos declined statistically from 53.7% at 1 week postoperative to 18.5% at 6 months postoperative and 7.4% at 12 months postoperative. Also, there were 16 patients (29.6%) with severe lid lag at 6 months and 5 (9.3%) at 12 months postoperatively ( Figs. 4 and 5 ).

The C-shaped frontalis flap suspension has certain limitations. First, it results in two scar lines, with the eyebrow scar being particularly visible, which may affect cosmetic outcomes. Additionally, the procedure requires more time to carefully avoid damaging the supraorbital nerve, which increases surgical complexity. Achieving good aesthetic symmetry between both eyes can also be challenging due to variations in muscle strength. Apart from the surgical technique, this study has inherent limitations. As a single-institution retrospective study, it is subject to selection bias and limited generalizability. Additionally, the absence of a control group makes comparisons with other techniques more challenging. Future research should include prospective, multicenter studies with control groups to minimize bias and validate findings.

### Conclusion

Our analysis of the modified frontalis muscle flap suspension technique—achieved by shaping the frontalis flap into a C-shape—demonstrates its efficacy in the treatment of moderate to severe blepharoptosis, particularly in cases with poor levator function. This approach offers the potential for long-lasting results, as no recurrences have been reported thus far. Additionally, it demonstrates promise for patients who have previously undergone frontalis sling surgery.
